# Fear extinction requires Arc/Arg3.1 expression in the basolateral amygdala

**DOI:** 10.1186/1756-6606-7-30

**Published:** 2014-04-23

**Authors:** Kousuke Onoue, Daisuke Nakayama, Yuji Ikegaya, Norio Matsuki, Hiroshi Nomura

**Affiliations:** 1 Laboratory of Chemical Pharmacology, Graduate School of Pharmaceutical Sciences, The University of Tokyo, 7-3-1 Hongo, Bunkyo-ku, Tokyo 113-0033, Japan; 2 Center for Information and Neural Networks, Suita, Osaka 565-0871, Japan

**Keywords:** Arc/Arg3.1, Fear conditioning, Extinction, Amygdala

## Abstract

**Background:**

Prolonged re-exposure to a fear-eliciting cue in the absence of an aversive event extinguishes the fear response to the cue, and has been clinically used as an exposure therapy. Arc (also known as Arg3.1) is implicated in synaptic and experience-dependent plasticity. Arc is regulated by the transcription factor cAMP response element binding protein, which is upregulated with and necessary for fear extinction. Because Arc expression is also activated with fear extinction, we hypothesized that Arc expression is required for fear extinction.

**Findings:**

Extinction training increased the proportion of Arc-labeled cells in the basolateral amygdala (BLA). *Arc* was transcribed during latter part of extinction training, which is possibly associated with fear extinction, as well as former part of extinction training. Intra-BLA infusions of *Arc* antisense oligodeoxynucleotide (ODN) before extinction training impaired long-term but not short-term extinction memory. Intra-BLA infusions of *Arc* antisense ODN 3 h after extinction training had no effect on fear extinction.

**Conclusion:**

Our findings demonstrate that Arc is required for long-term extinction of conditioned fear and contribute to the understanding of extinction as a therapeutic manner.

## Findings

### Background

Excessive fear is related to the pathogenesis of psychiatric disorders such as post-traumatic stress disorder. Prolonged re-exposure to a fear-eliciting cue in the absence of aversive events reduces the fear response to the cue, and has been clinically used as an exposure therapy. De novo gene expression contributes to the consolidation of fear extinction. In fact, activation of the transcription factor cAMP response element binding protein (CREB) increases with fear extinction, and its blockade impairs fear extinction [1]. Inhibition of protein synthesis also impairs fear extinction [2].

Reduction of synaptic strength is proposed as a cellular mechanism for fear extinction [3], which is accompanied by reduced firing of amygdala neurons [4] and a decrease in the surface expression of α-amino-3-hydroxy-5-methyl-4-isoxazolepropionic acid receptors (AMPARs) [5]. These mechanisms are supported by experiments demonstrating that the disruption of AMPAR endocytosis impairs fear extinction [5,6].

The activity-regulated cytoskeletal-associated protein (Arc, also known as Arg3.1) is transcribed during robust neural activity [7,8] and is involved in the inhibition of synaptic strength [9]. Increased expression of Arc reduces AMPAR-mediated synaptic transmission by accelerating receptor endocytosis [10,11]. *Arc* transcription is regulated by CREB [12], which is upregulated with and necessary for fear extinction [1]. Because Arc expression is also activated with fear extinction [1], we hypothesized that Arc expression would be required for fear extinction. We chose the basolateral amygdala (BLA) as a target region because gene expression here is essential for fear extinction [1]. In this study, we tested whether Arc expression in the BLA is required for extinction of contextual conditioned fear.

## Results

### Arc expression is upregulated in the BLA after extinction training

**Figure 1:**
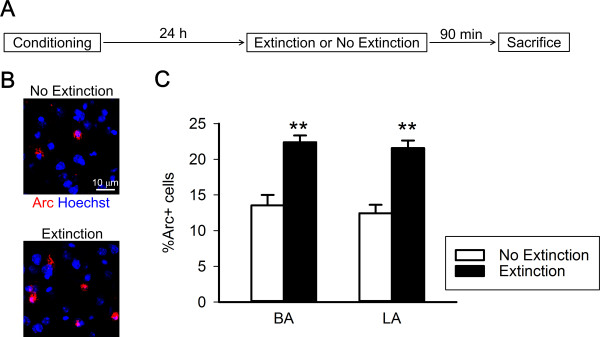
**Extinction training upregulates Arc expression in the BLA. (A)** The Extinction group underwent contextual fear conditioning and extinction training, in which they were re-exposed to the conditioning context for 40 min without shock. The No Extinction group underwent fear conditioning but not extinction training. **(B)** Representative images of Arc-immunolabeled cells in the Extinction and No Extinction groups, respectively. **(C)** The Extinction group demonstrated more Arc-labeled cells relative to the No Extinction group in both BA and LA (n = 6 mice per group, **p < 0.01).

In the first experiment, we tested whether extinction training upregulates Arc expression in the BLA. Mice in the Extinction group underwent contextual fear conditioning, and were subjected to extinction training the next day where they were re-exposed to the conditioning context for 40 min without shock (Experiment 1, Figure 1A). This long-term re-exposure decreased freezing duration over time (0–5 min vs. 35–40 min, *t*_(6)_ = 3.6, *p* = 0.016). Mice were killed 90 min after the extinction training, and their brains were processed immunohistochemically for Arc. Mice in the No Extinction group received contextual fear conditioning similarly to the Extinction group, but without extinction training. The Extinction group demonstrated more Arc-labeled cells relative to the No Extinction group, in both the basal (BA) (*t*_(10)_ = 4.3, *p* = 0.0016) and lateral amygdala (LA) (*t*_(10)_ = 4.8, *p* = 0.00069) (Figure 1B and C).

**Figure 2:**
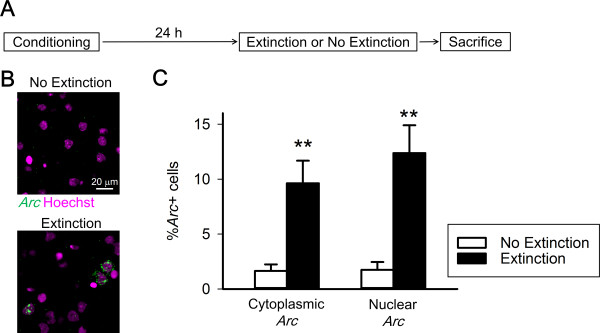
***Arc *is transcribed during both former and latter parts of extinction training. (A)** The Extinction group underwent contextual fear conditioning and extinction training, in which they were re-exposed to the conditioning context for 35 min without shock. The No Extinction group underwent fear conditioning but not extinction training. **(B)** Representative images of *Arc*-positive cells in the Extinction and No Extinction groups, respectively. **(C)** The Extinction group demonstrated more cytoplasmic and nuclear *Arc*-positive cells relative to the No Extinction group in the BLA (Extinction, n = 5 mice; No Extinction, n = 9 mice, **p < 0.01).

A previous study reported that even a few minutes of re-exposure to the conditioning context, which induces memory reconsolidation but not fear extinction, upregulates *Arc* expression [1,13]. Thus, greater Arc expression following extinction training might be related to memory reconsolidation but not fear extinction. To distinguish the Arc expression specific to the extinction training from that related to memory reactivation and/or reconsolidation, we utilized *Arc* cellular compartment analysis of temporal activity by fluorescence *in situ* hybridization (catFISH). Transcribed *Arc* RNA first appears in neuronal nuclei, and processed *Arc* mRNA then accumulates in the cytoplasm about 30 min after neural activity. Therefore, an analysis of the subcellular localization of *Arc* enables us to identify active neuronal ensembles during two time points that were separated by a 20–30 min interval. Mice in the Extinction group were re-exposed to the conditioning context for 35 min, and their brain slices were subjected to *Arc* fluorescence *in situ* hybridization (Experiment 2, Figure 2A). The Extinction group demonstrated more cytoplasmic and nuclear *Arc*+ cells relative to the No Extinction group in the basolateral amygdala (cytoplasmic *Arc*, *t*_(12)_ = 4.8, *p* = 0.00024; nuclear *Arc*, *t*_(12)_ = 5.2, *p* = 0.00024); Figure 2B and C). This result indicates that *Arc* transcription is induced during both former and latter parts of extinction training, and the latter part of *Arc* transcription is possibly associated with fear extinction but not memory reconsolidation.

### Inhibiting *Arc* translation in the BLA impairs long-term extinction of conditioned fear

**Figure 3:**
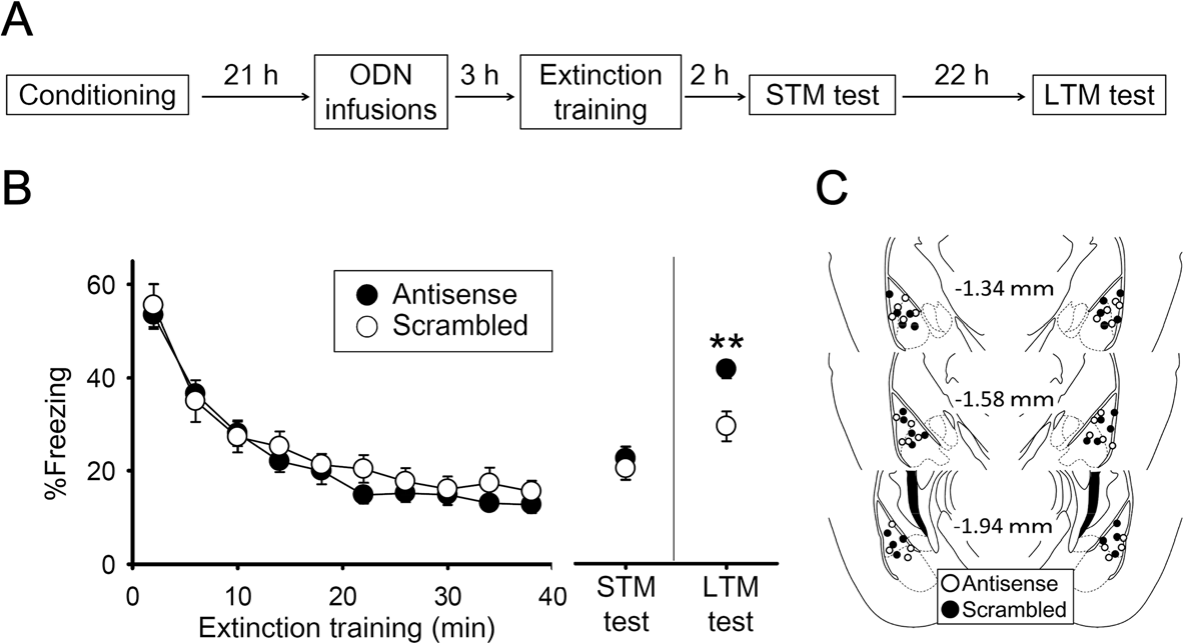
**Inhibiting *Arc *translation in the BLA impairs long-term extinction of conditioned fear. (A)** Mice received intra-BLA infusions of either *Arc* antisense or scrambled ODN 3 h before extinction training, and were subjected to short-term (STM) and long-term memory (LTM) tests 2 h and 24 h later, respectively. **(B)** Freezing time during extinction training decreased over time. The mice that received antisense ODN had a longer freezing period versus the mice that received scrambled ODN in the LTM test, but not the STM test (n = 14 mice per group, **p < 0.01). **(C)** Histological verification of cannula placements for mice infused with *Arc* antisense or scrambled ODN.

To inhibit Arc expression transiently, we employed antisense oligodeoxynucleotide (ODN) approach. By reference to a previous study [14], we designed *Arc* antisense ODN, which significantly decreased Arc levels but did not affect c-Fos or Zif268 levels (Additional file 1). Next, to ask whether Arc expression in the BLA is required for fear extinction, we inhibited Arc expression related to extinction training with the *Arc* antisense ODN. Mice underwent fear conditioning and extinction training (Experiment 4, Figure 3A). They received intra-BLA infusions of an *Arc* antisense or a scrambled ODN 3 h before extinction training (Figure 3C). Overall freezing duration did not differ across groups during extinction training (*F*_(1, 26)_ = 0.46, *p* = 0.51), and there was no significant group × time interaction (*F*_(9, 234)_ = 0.63, *p* = 0.77). Freezing duration did, however, decrease significantly over time (*F*_(9, 234)_ = 87.5, *p* = 8.2 × 10^−70^) (Figure 3B). Mice were subjected to short-term (STM) and long-term memory (LTM) tests 2 and 24 h later, respectively. Repeated-measures analysis of variance (ANOVA) revealed a significant group × test interaction (*F*_(1, 26)_ = 4.8, *p* = 0.038). The mice that received antisense ODN showed longer freezing relative to the mice that received scrambled ODN in the LTM test (*p* = 0.0028) but not the STM test (*p* = 0.54) (Figure 3B). These results indicate that Arc expression is required for long-term extinction of conditioned fear.

### Time-limited effect of inhibiting *Arc* translation in the BLA on fear extinction

**Figure 4:**
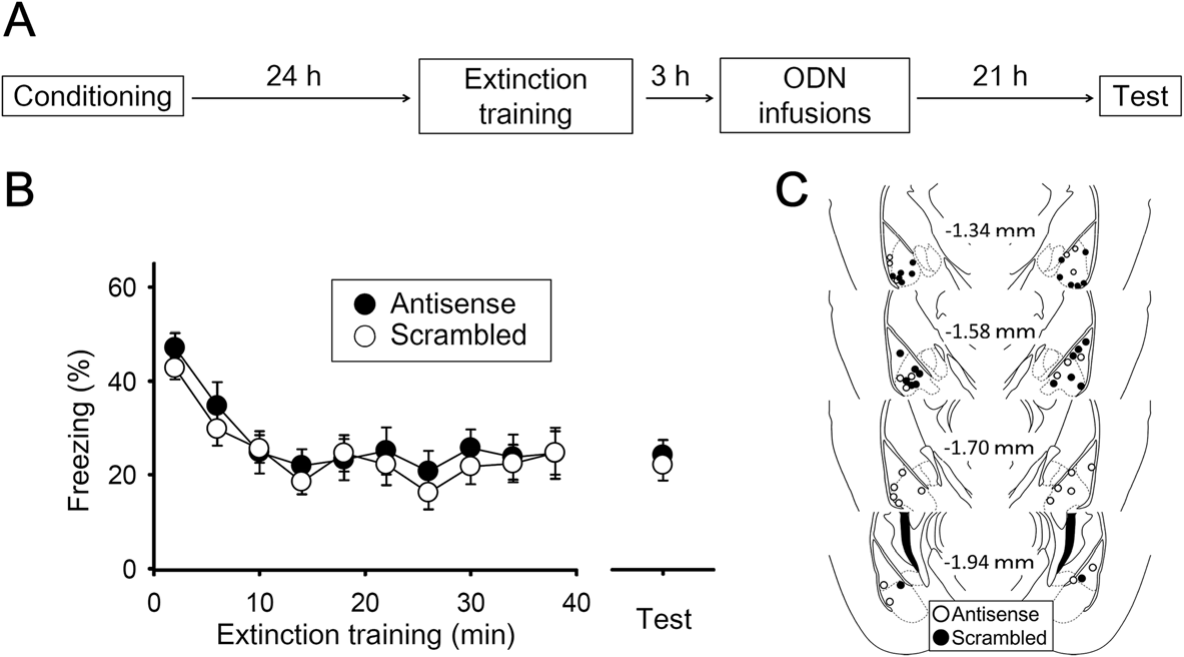
**Time-limited effect of inhibiting *Arc *translation in the BLA on fear extinction. (A)** Mice received intra-BLA infusions of either *Arc* antisense or scrambled ODN 3 h after extinction training and were given a memory test 24 h later. **(B)** Both groups exhibited equivalent periods of freezing (n = 13 mice per group). **(C)** Histological verification of cannula placements for mice infused with Arc antisense or scrambled ODN.

Finally, we tested whether the effect of inhibiting BLA *Arc* translation on fear extinction depends on the time interval between extinction training and Arc inhibition. Mice were subjected to both fear conditioning and extinction training (Experiment 5, Figure 4A). Freezing duration decreased over time with extinction training (time effect, *F*_(9, 216)_ =14.99, *p* = 7.78 × 10^−19^). Mice received intra-BLA infusions of either *Arc* antisense ODN or scrambled ODN 3 h after extinction training (Figure 4C). On the next day, both groups exhibited equivalent periods of freezing (*t*_(24)_ = 0.30, *p* = 0.77) (Figure 4B), suggesting that the effect of inhibiting *Arc* translation in the BLA on fear extinction is time-limited.

## Discussion

In this study, we have demonstrated the involvement of Arc in fear extinction. We found that 1) extinction training increased the proportion of Arc-labeled cells in the BLA; 2) *Arc* was transcribed during latter part as well as former part of extinction training; 3) intra-BLA infusions of *Arc* antisense ODN before extinction training impaired long-term but not short-term extinction memory; and 4) intra-BLA infusions of *Arc* antisense ODN 3 h after extinction training had no effect on fear extinction. Collectively, these findings indicate that de novo Arc expression, induced by extinction training, contributes to long-term extinction of contextual conditioned fear.

Our study design suggests that this impaired extinction of conditioned fear cannot be explained by a nonspecific effect of intra-BLA infusions of *Arc* antisense ODN. First, the Arc antisense ODN effectively inhibited Arc expression and exhibited a high degree of specificity for *Arc* relative to other immediate-early genes (IEGs) (Additional file 1). Second, the behavior of the mice that received *Arc* antisense ODN during extinction training and the short-term memory test was comparable to that of the mice that received scrambled ODN, indicating that the antisense ODN had no effect on retrieval of fear memory and acquisition of extinction memory. More importantly, fear extinction in the mice that received *Arc* antisense ODN 3 h after extinction training was intact, indicating that extinction training-induced *Arc* translation is essential for fear extinction.

Arc may contribute to fear extinction by reducing synaptic strength. Fear memory is thought to be maintained by fear conditioning-induced potentiation of synaptic efficacy in amygdalar synapses [15,16]. Extinction training reverses this potentiation through accelerated endocytosis of AMPAR [5]. This endocytosis-regulated depotentiation is likely an essential mechanism for fear extinction, because inhibiting AMPAR endocytosis impairs fear extinction [5,6]. In this study, we found that Arc expression is required for fear extinction. Arc interacts with endophilin and dynamin to modulate AMPAR endocytosis [11], thus allowing Arc to influence synaptic strength and homeostasis [9,17]. Taken as a whole, extinction training induces Arc expression, which possibly accelerates AMPAR endocytosis and reverses fear conditioning-induced synaptic potentiation. The resulting depotentiation is likely to contribute to fear extinction.

In short, our findings provide evidence that Arc is critical for long-term extinction of conditioned fear, and that activation of Arc signaling in the BLA might lead to long-term extinction of fear memory through reversal of fear conditioning-induced synaptic potentiation. A better understanding of the mechanisms underlying fear extinction is necessary for the development of new treatments for psychiatric disorders such as post-traumatic stress disorder. These data suggest that a BLA Arc-regulated pathway could generate viable targets for these potential new therapies.

## Materials and methods

### Animal experiment ethics

All experiments were approved by the animal experiment ethics committee at the University of Tokyo (approval number 24–10), and were in accordance with the University of Tokyo guidelines for the care and use of laboratory animals.

### Subjects

Male C57BL/6J mice (Japan SLC Inc., Shizuoka, Japan), weighing 20–30 g and aged 8–13 weeks, were housed 2–4 per cage, and kept on a 12-h light/dark cycle (lights on from 7:00 a.m. to 7:00 p.m.). They were given free access to food and water, and acclimated to daily handling for 1 week prior to the start of the study.

### Behavioral procedure

Contextual fear conditioning, extinction training, and subsequent testing were performed in a conditioning chamber (17 cm wide, 15 cm deep, and 15 cm high) with a stainless steel grid floor (O’ Hara & Co., Ltd, Tokyo, Japan). A conditioning session consisted of placing the animal in the chamber and delivering a 2-s foot shock (0.3 mA) after 130 s. The mice then received 2 additional shocks every 100 s. They were kept in the chamber for an additional 50 s and were then returned to their home cage. For extinction training and testing, mice were re-exposed to the conditioning chamber without shock. All sessions were performed between 9:00 a.m. and 1:00 p.m., and each session was video-recorded for automatic scoring of freezing as described previously [18].

In Experiment 1, mice in the Extinction group underwent 40 min of extinction training 24 h after fear conditioning and were killed 90 min later. Mice in the No Extinction group received fear conditioning, and 1 day later, they were killed immediately following removal from the home cage. Their brain slices were subjected to Arc immunohistochemistry.

In Experiment 2, mice in the Extinction group underwent 35 min of extinction training 24 h after fear conditioning and were killed immediately after extinction training. Their brain slices were subjected to *Arc* fluorescent *in situ* hybridization.

In Experiment 3, we examined the effect and specificity of *Arc* antisense ODN. Mice received *Arc* antisense or scrambled ODN infusions into the hippocampus and underwent fear conditioning 1.5 or 3 h later. Mice were decapitated after diethyl ether anesthesia 2 h after fear conditioning, and their brains were rapidly removed and frozen at −80°C. Coronal brain sections (300 μm) were prepared using a cryostat. The dorsal hippocampus (1.30 to 1.90 mm posterior to bregma) was punched out and homogenized in RIPA buffer (08714–04, Nacalai Tesque, Kyoto, Japan).

In Experiment 4, mice underwent extinction training 24 h after fear conditioning. To determine if Arc inhibition affected fear extinction, mice received intra-BLA infusions of either *Arc* antisense or scrambled ODN 3 h before extinction training, and were subjected to a short-term and long-term memory tests 2 h and 24 h later, respectively.

In Experiment 5, mice underwent extinction training 24 h after fear conditioning. To assess the effect of the post-extinction training time interval, mice received intra-BLA infusions of either *Arc* antisense or scrambled ODN 3 h after extinction training and were subjected to a long-term memory test 24 h later.

### Microinfusions

Surgery for intra-BLA infusions was conducted according to our previous study [19]. Briefly, mice were anesthetized with pentobarbital (2.5 mg/kg, i.p.) and xylazine (10 mg/kg, i.p.), and 26-gauge stainless-steel guide cannulae (Plastics One, VA, USA) were implanted in the BLA (AP = −1.4, ML = ±3.5, DV = −4.8 mm relative to bregma). Mice were given at least 7 days of postoperative recovery time. Microinfusions of *Arc* antisense or scrambled ODN (400 pmol; 0.5 μL/side) were made over 2 min, and the infusion cannulae were left in place for at least 2 min afterwards in order to facilitate the diffusion of ODN throughout the whole BLA.

*Arc* antisense ODN and scrambled ODN (GeneDesign Inc, Osaka, Japan) were designed in reference to a previous study [14]. The *Arc* ODN encoded an antisense sequence for the *Arc* mRNA sequence near the translation start site [7]. The scrambled ODN does not show significant homology to any sequences in the GenBank database [14]. Both ODNs contained phosphorothioate linkages on the three terminal bases of both the 5′ and 3′ ends, and phosphodiester internal bonds. This design is reported more stable than unmodified phosphorothioated ODNs in vivo, and less toxic than fully phosphorothioated ODNs [14]. The following sequences were used (“~” denotes a phosphorothioate linkage): 5′-G~T~C~CAGCTCCATCTGGT~C~G~T-3′ (antisense) and 5′-C~G~T~GCACCTCTCGCAGG~T~T~T-3′ (scrambled).

In the Experiment 3, the guide cannulae were implanted in the dorsal hippocampus (−2.0, ±1.7, −2.1 mm relative to bregma).

### Immunohistochemistry and image analysis

After the anesthetization, mice were transcardially perfused with PBS followed by 4% paraformaldehyde (PFA). Brains were post-fixed in 4% PFA for 12 h. Free-floating coronal sections (40 μm) were prepared using a cryostat (HM520; Thermo Fisher Scientific, Waltham, MA, USA). Arc was visualized with anti-Arc primary antibody (1:1,000; SySy, Gottingen, Germany), biotinylated anti-rabbit secondary antibody (1:500; Vector Laboratories, Burlingame, CA, USA), VECTASTAIN ABC Kit (Vector Laboratories), and Cy3-tyramide (1:100; Perkin-Elmer, Waltham, MA, USA). Nuclei were counterstained with Hoechst dye (1:1000; Invitrogen).

Images of the amygdala were acquired by z-stacks using a confocal microscope (CV1000; YOKOGAWA, Tokyo, Japan) at 40 × under an oil-immersion lens (NA 1.3). Images of BA and LA from three sections (1.34, 1.58, 1.82 mm posterior to bregma) per mouse were analyzed using ImageJ (NIH).

### Fluorescent *in situ* hybridization and image analysis

After the mice were sacrificed, the brains were frozen quickly and stored at −80°C until further processing. Brains were sectioned (20 μm) with a cryostat, mounted on slides and stored at −80°C. Antisense riboprobes for *Arc*, conjugated to digoxigenin-UTP (Roche Applied Science, Penzberg, Germany), were generated from cDNA plasmid (provided by Dr. Paul F Worley) containing an almost full-length cDNA of the *Arc* transcript using MAXIScript (Ambion, Austin, TX USA). *In situ* hybridization was performed according to previously published protocols [20]. Slide-mounted brain sections were fixed in 4% PFA, acetylated with 0.5% acetic anhydride/1.5% triethanolamine, dehydrated through 50% methanol/50% acetone solutions, and equilibrated in 2× saline sodium citrate buffer (SSC). Slides were incubated in prehybridization buffer for 30 min. The antisense riboprobe was diluted to 150 μL in hybridization buffer, heat denatured, chilled on ice, and applied to each slide. Hybridization was carried out at 56°C for 16 h. Slides were washed to a final stringency of 0.5×SSC at 56°C, which included an earlier wash step at 37°C in 2×SSC with RNase A (10 μg/mL). Endogenous peroxidase activity was quenched with 2% H_2_O_2_ in 1×SSC. Slides were blocked with TSA blocking reagent (PerkinElmer, Waltham, MA, USA) and incubated with an anti-digoxigenin horseradish peroxidase conjugate (1:500, Roche) for 2 h. Slides were washed three times in Tris-buffered saline with 0.05% Tween-20, and incubated with fluorescein-tyramine working solution (1:10, PerkinElmer). Slides were washed in PBS and the nuclei were counterstained with propidium iodide (PI, 10 μM; Life Technologies) for 10 min. Finally, the sections were washed with PBS and mounted in Permafluor (Thermo Fisher Scientific, Waltham, MA, USA).

Images of the BLA (1.58 mm posterior to bregma) were acquired by collecting z-stacks using a confocal microscope (LSM-510; Zeiss, Oberkochen, Germanay) at 40× under an water-immersion lens (NA 1.2) and analyzed using in-house Matlab program. From 4 to 8 sections per mouse were counted for the analysis. Only cells that contained whole nuclei and were presumptive neurons, with large nuclei stained diffusely with propidium iodide, were included in the analysis. The designation “nuclear *Arc* positive” was assigned to neurons that exhibited 1 or 2 of the characteristic intense intranuclear areas of fluorescence; the designation “cytoplasmic *Arc* positive” was assigned to neurons that contained perinuclear/cytoplasmic labeling over multiple optical sections. Labels were assigned by an experimenter blind to behavioral conditions.

### Western blotting

Protein concentrations were normalized across homogenates using a Bradford assay. Equal amounts of protein were electrophoresed on 5-20% SDS polyacrylamide gels and transferred to PVDF membranes. Western blots were blocked in blocking buffer (03953–95, Nacalai Tesque) and then incubated with an anti-*beta*-actin antibody (1:1000; A5441, Sigma), an anti-Arc/Arg3.1 antibody (1:1000; sc-17839, Santa Cruz Biotechnology), anti-c-Fos antibody (1:100; Ab-2, Calbiochem) or anti-Zif268 antibody (1:1000; #4153, Cell Signaling). After incubation with anti-mouse IgG (1:100000; A9044, Sigma) or anti-rabbit IgG (1:10000; 01827–44, Nacalai Tesque), bands were developed with a chemiluminescent substrate (RPN2132, GE Healthcare). The immunopositive signals were detected by ImageQuant LAS 4000 (GE Healthcare) and analyzed using ImageJ (NIH).

### Data analysis

All values were reported as mean ± SEM. Statistical analysis was performed using Student’s *t*-test, repeated-measures ANOVA, one-way ANOVA and Tukey’s test.

## Abbreviations

Arc: Activity-regulated cytoskeletal-associated protein; CREB: cAMP response element binding protein; AMPAR: α-amino-3-hydroxy-5-methyl-4-isoxazolepropionic acid receptor; BLA: Basolateral amygdala; BA: Basal amygdala; LA: Lateral amygdala; STM: Short-term memory; LTM: Long-term memory; ODN: Oligodeoxynucleotide; IEGs: Immediate-early genes; PFA: Paraformaldehyde; catFISH: cellular analysis of temporal activity by fluorescence *in situ* hybridization.

## Competing interests

The authors declare that they have no competing interests.

## Authors’ contributions

KO, DN and HN performed experiments and analyzed the data. DN and HN designed the study. KO and HN wrote the manuscript. NM and YI supervised the project. All authors read and approved the final manuscript.

## Supplementary Material

Additional file 1 ***Arc *antisense ODN infusions inhibit Arc expression but not c-Fos or Zif268 expression.** Mice were infused with Arc antisense or scrambled ODN 1.5 h or 3 h before conditioning. Mice were killed 2 h after conditioning. Control mice received fear conditioning without infusions. Arc, c-Fos and Zif268 levels were normalized with the level of control mice. **(A)***Arc* antisense ODN infusions 3 h but not 1.5 h before conditioning decreased Arc levels (One-way ANOVA, *F*_(2,15)_ = 10.8, *p* = 0.0012; post-hoc Tukey’s test, Scrambled vs. Antisense (3 h), *p* = 0.0017). **(B, C)** These infusions did not affect c-Fos or Zif268 levels (c-Fos, *t*_(10)_ = 0.91, *p* = 0.38; Zif268,*t*_(10)_ = 0.85, *p* = 0.41). **p < 0.01. Click here for file
