# Supplementary figures and images for: Fear extinction requires Arc/Arg3.1 expression in the basolateral amygdala
Source: Mol Brain. 2014 Apr 23;7:30. doi: 10.1186/1756-6606-7-30 (PMC4022082; doi:10.1186/1756-6606-7-30)

A

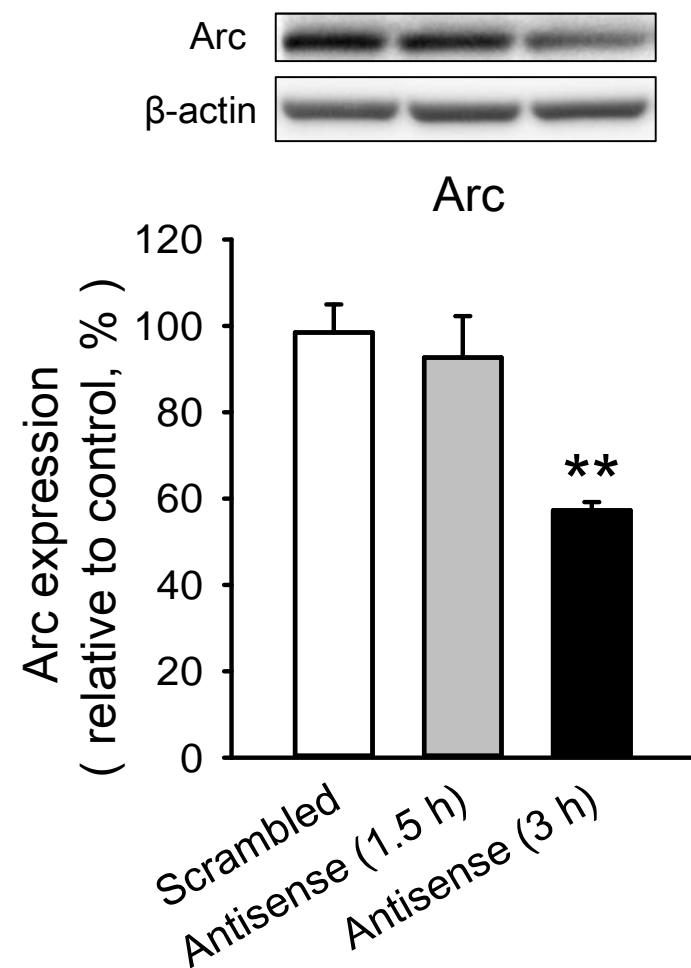

B

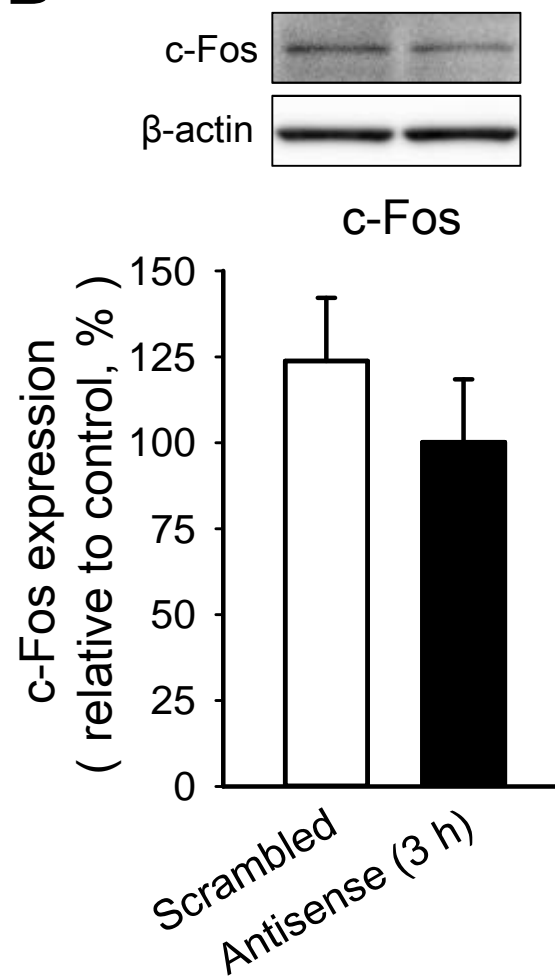

C

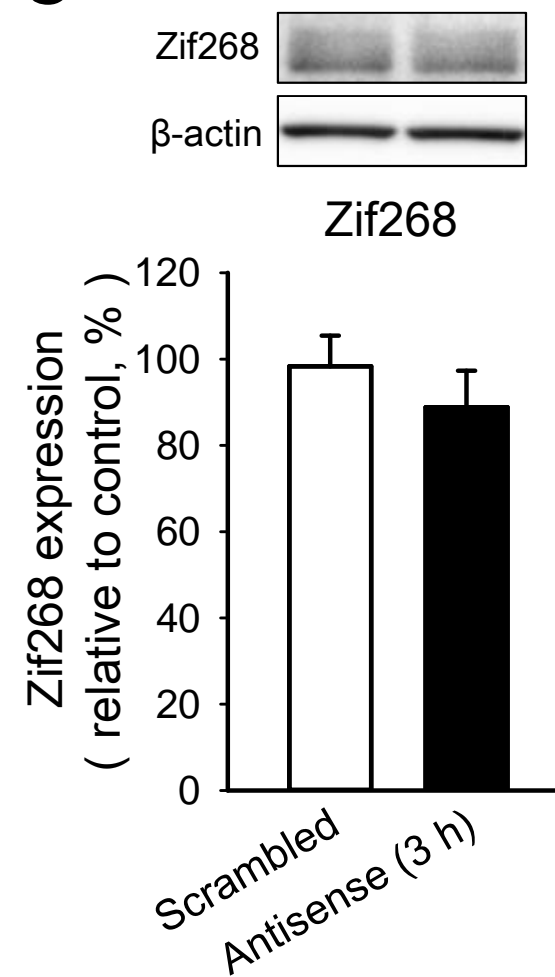

Supplement: Additional file 1 — Arc antisense ODN infusions inhibit Arc expression but not c-Fos or Zif268 expression. Mice were infused with Arc antisense or scrambled ODN 1.5 h or 3 h before conditioning. Mice were killed 2 h after conditioning. Control mice received fear conditioning without infusions. Arc, c-Fos and Zif268 levels were normalized with the level of control mice. (A)Arc antisense ODN infusions 3 h but not 1.5 h before conditioning decreased Arc levels (One-way ANOVA, F(2,15) = 10.8, p = 0.0012; post-hoc Tukey’s test, Scrambled vs. Antisense (3 h), p = 0.0017). (B, C) These infusions did not affect c-Fos or Zif268 levels (c-Fos, t(10) = 0.91, p = 0.38; Zif268,t(10) = 0.85, p = 0.41). **p < 0.01. [file 1756-6606-7-30-S1.pdf]
